# Exploring a peptide nucleic acid-based antisense approach for CD5 targeting in chronic lymphocytic leukemia

**DOI:** 10.1371/journal.pone.0266090

**Published:** 2022-03-31

**Authors:** Elena Cesaro, Andrea Patrizia Falanga, Rosa Catapano, Francesca Greco, Simona Romano, Nicola Borbone, Arianna Pastore, Maria Marzano, Federico Chiurazzi, Stefano D’Errico, Gennaro Piccialli, Giorgia Oliviero, Paola Costanzo, Michela Grosso

**Affiliations:** 1 Department of Molecular Medicine and Medical Biotechnology, University of Naples Federico II, Napoli, Italy; 2 Department of Pharmacy, University of Naples Federico II, Napoli, Italy; 3 ISBE-IT, University of Naples Federico II, Napoli, Italy; 4 Department of Clinical and Experimental Medicine, Division of Hematology, University of Naples Federico II, Napoli, Italy; University of Manitoba, CANADA

## Abstract

We herein report an innovative antisense approach based on Peptide Nucleic Acids (PNAs) to down-modulate CD5 expression levels in chronic lymphocytic leukemia (CLL). Using bioinformatics tools, we selected a 12-mer tract of the CD5 mRNA as the molecular target and synthesized the complementary and control PNA strands bearing a serine phosphate dipeptide tail to enhance their water solubility and bioavailability. The specific recognition of the 12-mer DNA strand, corresponding to the target mRNA sequence by the complementary PNA strand, was confirmed by non-denaturing polyacrylamide gel electrophoresis, thermal difference spectroscopy, circular dichroism (CD), and CD melting studies. Cytofluorimetric assays and real-time PCR analysis demonstrated the downregulation of CD5 expression due to incubation with the anti-CD5 PNA at RNA and protein levels in Jurkat cell line and peripheral blood mononuclear cells from B-CLL patients. Interestingly, we also observed that transfection with the anti-CD5 PNA increases apoptotic response induced by fludarabine in B-CLL cells. The herein reported results suggest that PNAs could represent a potential candidate for the development of antisense therapeutic agents in CLL.

## Introduction

B-cell chronic lymphocytic leukemia (B-CLL), the most frequent type of leukemia in adults, is characterized by the clonal expansion of mature CD5+ B-lymphocytes that accumulate in peripheral blood, bone marrow, and lymphatic tissues [1, 2]. CD5 is a surface glycoprotein expressed in normal T-lymphocyte and only in a small subset of B-lymphocytes. Additionally, its aberrant expression is detected in some B-lymphocyte malignancies, including B-CLL, for which immunophenotyping diagnostic protocols include detection of cell surface CD5 antigene [3]. Recently, CD5 expression levels have also been proposed as a novel prognostic marker [4–6]. The presentation and course of CLL are highly variable. In some cases, the disease is latent, whereas in others, it rapidly progresses with an aggressive course. Despite significant progress in therapy options, the more severe conditions are characterized by an inadequate response to conventional treatment and the development of drug resistance. Accordingly, novel treatments efficiently directed toward specific CLL targets still lack and represent an urgent medical need. Recent compelling evidence suggests that CD5 abnormal expression is involved in the development and progression of B-CLL through negative regulation of BCR-induced signaling or signaling pathways triggered by CD5 itself [7–9]. Therefore, besides representing a diagnostic and prognostic marker, CD5 is emerging as a promising therapeutic target in B-CLL [10]. CD5 is highly expressed in B-CLL cells, but it is almost undetectable in normal B-cells, thus supporting a role of CD5 as a specific target for B-CLL. In the light of this evidence, many preclinical studies are being conducted to explore the potential use of anti-CD5 immunotherapy approaches in B-CLL treatment [11, 12]. In addition, the inhibition of CD5 expression by antisense strategies based on natural and modified oligonucleotides (ONs) could represent a valid alternative approach to be explored [13]. With this aim, in this paper we evaluated the feasibility of an antisense Peptide Nucleic Acid (PNA)-based approach to target CD5. PNAs are synthetic analogs of DNA and RNA in which the canonical sugar-phosphate backbone is replaced by an N-(2-aminoethyl)-glycine repeating unit [14]. PNAs represent an attractive tool to selectively modulate gene expression using either antisense [15–17], anti-miRNA [18–20], or antigene [21, 22] strategies.

The study reported in this paper can be divided into two parts. In the first part, we investigated by chemical-physical methodologies the ability of the synthesized 12-mer PNA to specifically recognize the complementary ON sequence located in the target mRNA. Non-denaturing polyacrylamide gel electrophoresis (PAGE), thermal difference spectra (TDS), circular dichroism (CD), and CD melting studies allowed us to ascertain the topology of the obtained complex, as well as the hybridization efficiency and structural stability. In the second part, we used an immortalized human leukemia T-cell line (Jurkat cells), which stably expresses the CD5 protein at high levels, and peripheral blood mononuclear cells (PBMC) from B-CLL patients to assess the ability of PNA to down-regulate the expression of CD5 as intended. In Jurkat cells, we demonstrated that PNA decreased CD5 expression both at mRNA and protein levels. To determine the consequences of PNA-mediated CD5 down-modulation in a more physiologically relevant setting, we carried out PNA treatment in PBMCs isolated from B-CLL patients. Consistent with results obtained in Jurkat cells, we observed CD5 reduction following PNA treatment. Intriguingly, we observed that PNA co-treatment with fludarabine significantly increases the drug-induced apoptotic effects, thus possibly paving the way to the development of novel therapeutic strategies.

## Materials and methods

### DNA analysis, synthesis, and characterization

To select and design an efficient antisense PNA targeting the CD5 mRNA sequence (NCBI Reference Sequence: NM_014207.4), we used the OligoWalk algorithm embedded in the RNA structure software (http://rna.urmc.rochester.edu/). The M-fold algorithm (http://rothlab.ucdavis.edu/genhelp/mfold.html) was also used to predict secondary structures in CD5 mRNA to exclude those sequences that could fall back on not accessible mRNA regions. Among all the selected 12-mer putative targets on the CD5 mRNA, we chose a tract with a high purine base content, starting 1050 nt from ATG, because of the higher synthetic yield of pyrimidine-rich PNA strands. The synthesis, purification, and desalting of the corresponding DNA strand (**DNA**, Table 1) were performed by standard methods following the protocols reported elsewhere [22]. The ON concentration was determined using a Jasco (Easton, MD, USA) V-530 UV spectrophotometer at 260 nm and 90°C, using the molar extinction coefficient ε = 131.6 cm^–1^ mM^–1^, calculated with the Sigma-Aldrich OligoEvaluatorTM web tool (www.oligoevaluator.com). The structure of **DNA**, dissolved in ammonium acetate buffer and methanol (1:1, v/v) at the final concentration of 2 μM, was confirmed by ESI-MS (S1 Fig). The 12-mer purine-rich control DNA and pyrimidine-rich control DNA sequences (Table 1) were synthesized and purified as previously described.

**Table 1:** PNA and DNA molecules used in this study.

Sample	Sequence
**PNA**	tttctctcccaa-Gly-Ser(P)-Ser(P)-Gly-NH_2_ (N→C)
**PNA-FITC**	FITC(AEEA)_2_-tttctctcccaa- Gly-Ser(P)-Ser(P)-Gly-NH_2_ (N→C)
**scrambled PNA**	cctattactcct-Gly-Ser(P)-Ser(P)-Gly-NH_2_ (N→C)
**scrambled PNA-FITC**	FITC(AEEA)_2_-cctattactcct-Gly-Ser(P)-Ser(P)-Gly-NH_2_ (N→C)
**DNA**	TTGGGAGAGAAA (5’→3’)
**pyrimidine-rich control DNA**	CCTCTGGTCTCC (5’→3’)
**purine-rich control DNA**	GGAGACCAGAGG (5’→3’)

Lowercase letters indicate PNA bases.

### PNA synthesis and characterization

Specific CD5 PNA (**PNA**) and a **scrambled PNA** (Table 1) used as non-specific negative control were synthesized using the 9-fluorenylmethoxycarbonyl (Fmoc) solid-phase strategy and purified following the protocol reported elsewhere [23].

To promote the PNAs delivery across the cellular membranes, we used Lipofectamine 2000 (Invitrogen, Carlsbad, CA) as a transfection reagent. The cationic head group of lipofectamine governs the interaction between lipids and PNAs. Due to the absence of charges in the PNA backbone, it was necessary to introduce two negative charges on the PNA oligomers by adding two serine-phosphate monomers at the C-end of the PNA chains. For this purpose, we used two Fmoc-Gly-OH and two Fmoc-L-Ser [PO(OBzl) OH]-OH residues in the first four couplings of the solid-phase strategy. To evaluate PNAs’ transfection efficiency and select the transfected cells, we labeled the PNAs’ N-terminus with the fluorescein isothiocyanate (FITC) fluorophore, thus obtaining the corresponding **PNA-FITC** and **scrambled PNA-FITC** (Table 1). After purification by RP-HPLC, all the PNA products were characterized by ESI-MS (S2 Fig). The amount of all PNA samples, dissolved in pure water, was estimated by a Jasco V-530 UV spectrophotometer at 260 nm and 90°C, using the molar extinction coefficient ε = 104.4 cm^–1^ mM^–1^, calculated with the Sigma-Aldrich OligoEvaluatorTM web tool (www.oligoevaluator.com).

### Preparation of samples

All DNA and PNA samples were analyzed in 100 mM phosphate-buffered saline (PBS) at pH = 6.8. For DNA and PNA preparations, 10 nmol of each ON were lyophilized and dissolved in 10 μL of 100 mM PBS buffer to obtain 1 mM stock solutions. DNA/PNA mixtures were prepared at the 1:3 ratio by mixing 10 nmol of lyophilized DNA with 30 μL of 1 mM PNA stock solution in water. Sample solutions were dried and re-dissolved in 10 μL of PBS to have 1 mM solutions. Finally, solutions were heated at 90°C for 10 min, equilibrated at 4°C overnight, and used for PAGE analysis. For CD studies, 7 μL of each sample were diluted to 400 μL with 100 mM PBS buffer to obtain a 17.5 μM concentration. These solutions were further diluted to 3.5 μM for TDS investigations. Finally, a 50 μM solution of **DNA/PNA** mixture was used for CD melting measurement.

### Non-denaturating Polyacrylamide Gel Electrophoresis (PAGE)

Polyacrylamide gel was prepared at 18% of acrylamide/bis-acrylamide solution. 1 × Tris-Borate-EDTA (TBE) buffer supplemented with 30 mM KCl at pH 7.0 was employed for the gel run. Samples were loaded at 1 mM concentration. 3 μL of each sample was added to 7 μL of loading buffer (glycerol/1 × TBE + 30 mM KCl 1:9) for gel loading. PAGE was carried out at a constant voltage of 120 V at 5°C for about 1 h. The gel was visualized by using a UV-Vis lamp at 254 nm.

### Thermal Difference Spectra (TDS)

TDS of **DNA/PNA** and **DNA/scrambled PNA** were obtained by the arithmetic difference between UV spectra acquired at 90°C (unfolded) and 5°C (folded). The UV spectra were recorded at a concentration of 3.5 μM of samples on a Jasco V-530 UV spectrophotometer equipped with a Peltier-type temperature control system (model PTC348WI) using the following settings: range λ = 250–320 nm, 400 nm min^–1^ scanning speed, 2.0 nm bandwidth, and averaged over three scans using 0.1 cm path-length cuvette.

### Circular Dichroism (CD) and CD melting

CD spectra were recorded at 5°C using a Jasco 1500 spectropolarimeter equipped with a Jasco PTC-348-WI temperature controller unit. The thermal denaturation curve of **DNA/PNA** mixture was recorded at 265 nm in the temperature range 5–90°C, 1°C min^–1^ heating rate.

### Peripheral Blood Mononuclear Cells (PBMCs) isolation from CLL patients and drug treatment

Peripheral Blood Mononuclear Cells (PBMCs) were isolated by a Ficoll-PaqueTM density gradient (Merck, Darmstadt, Germany) from the peripheral blood of B-CLL untreated patients in the stationary phase of the disease [24]. B-CLL diagnosis was obtained according to clinical and immunophenotypic criteria. Five patients (P1, P2, P3, P4, P5) who had > 75% CD19+ cells co-expressing CD5 (patients P1-P5) were selected. The patients provided appropriate written informed consent. The study was approved by the Ethics Committee of the University of Naples Federico II. PBMCs were maintained in RPMI 1640 (Sigma-Aldrich, Milan, Italy) supplemented with 10% human serum and 20 μL of anti-Human CD3 antibody (10 μg/mL) (eBioscience Thermo Fisher, Inc, Waltham, MA) as previously reported [25, 26]. For drug treatment, 24 h after PNA transfection, PBMCs were treated with 9 μM fludarabine (Teva Pharmaceutical Industries Ltd, UK) for 72 h.

### Cell cultures and treatment

The human Jurkat cell line was obtained from the Cell Culture Facility, CEINGE (Naples, Italy). Cells were maintained in RPMI 1640 (Sigma) medium supplemented with 10% fetal bovine serum (FBS) (Gibco, Thermo Fisher Scientific Inc, Waltham, MA) at 37°C in a humidified 5% CO_2_ atmosphere. As a positive control of cell death, Jurkat cells were treated with 20 μM cisplatin (Accord Healthcare, London, UK) for 24 h.

### Transient transfection

For transfection experiments, freeze-dried PNAs were dissolved in RNase-free water and trifluoroacetic acid (TFA). Jurkat cells were plated in 12-well plates at a density of 4 × 10^5^ cells/well and transiently transfected with either 1 μM or 2.5 μM **PNA** or **scrambled PNA** as an aspecific negative control, using Lipofectamine 2000 (Invitrogen, Carlsbad, CA) as transfection reagent as previously reported [27, 28]. Also, CLL PBMCs were plated in 12-well plates at a density of 1 × 10^6^ cells/well and transiently transfected with 1 μM **PNA** or **scrambled PNA**. 48 h after transfection, Jurkat cells, and CLL PBMCs were collected to evaluate CD5 mRNA and protein levels. The same cells were also used to perform Annexin V/propidium iodide (PI) assays.

### Real-time PCR analysis

Total RNA extraction from Jurkat cells and B-CLL PBMCs, reverse transcription, and quantitative real-time PCR were performed as previously described [29–31]. Primers used to detect the expression of CD5, and HPRT (endogenous control) were:

CD5 (sense): 5′‐CAGCCTCCCACGTGGATAAC‐3′,

CD5 (antisense): 5′‐TCAGGACAAACAGGTCTGGC‐3′,

HPRT (sense): 5′‐TGACACTGGCAAAACAATGCA‐3′,

HPRT (antisense): 5′-CCACCACTGCATCAAATTCATG‐3′

Each real-time PCR was performed in triplicate in a 15 μL reaction mix containing 7.5 μL of 2 × SsoAdvanced Universal SYBR Green supermix (Bio-Rad Laboratories GmbH, Munich, Germany), 0.28 μL of a 20 μM primer mix, 1.5 μL of cDNA, and 5.72 μL of nuclease-free water. The cycling parameters were set up as follows: a denaturation step at 95°C for 3 min, followed by 40 cycles (95°C for 15 s, 60°C for 30 s) and 80 cycles performed according to standard protocols for melting curve analysis.

### Flow cytometric analysis

To evaluate the transfection efficiency, 1 × 10^5^ Jurkat cells were harvested 48 h after transfection with **PNA-FITC and scrambled PNA-FITC**, washed in PBS, and analyzed with a BD AccuriTM C6 Cytometer (BD Biosciences, San Jose, CA, USA). The study population was identified and gated on the base of its forward and side scatter to exclude debris found at the bottom left corner of the FSC/SSC density plot. Gated events, of the acquired population, were then analyzed for their fluorescence in the FL-1 channel to assess PNA delivery thanks to their labeling to the FITC fluorochrome. Data were expressed in a FL-1 histogram where, in order to accurately identify the positive dataset, non-transfected cells were used to place a vertical bar and determine the background/autofluorescence and to set the negative population, thus allowing the positive cells to be accurately identified and gated for further analysis.

Membrane CD5 staining in Jurkat cells was performed using anti-CD5-phycoerythrin (PE)-conjugated (ImmunoTools, Friesoythe, Germany) at a concentration of 0.05 μg/mL. Instead, the intracellular CD5 expression was measured on Jurkat cells fixed with 1% paraformaldehyde in PBS 1× for 20 min and permeabilized with 0.5% Triton X-100. Cells were then incubated with the specific anti-CD5 antibody PE-conjugated. B-CLL PBMCs were identified using an FSC/SSC dual parameter dot plot and characterized for the expression of CD19 and CD5. The measure of membrane CD5 and CD19 was performed using an anti-CD5 Allophycocyanin (APC)-conjugated and anti-CD19 (FITC)-conjugated (ImmunoTools), at a concentration of 0.05 μg/mL. Human Fc block (2.5 μg/10^6^ cells) (Pharmingen BD, San Diego, CA, USA) was used to minimize the non-specific binding of immunoglobulins to Fc receptors before the flow cytometric staining. Control IgG isotypes conjugated with each used fluorochrome, IgG-PE/FITC (Santa Cruz Biotechnology, Santa Cruz, CA, USA), or APC-conjugated (Pharmingen BD) were used in each staining to assess the non-specific binding. Each staining was performed by incubating cells with the antibodies mentioned above for 30 min in the dark at 4°C. Cells were then washed and analyzed by flow cytometry. Cell death analysis was conducted by double-staining with Annexin-V (FITC-conjugated, Immunotools) and Propidium iodide (PI) (Merck Millipore, Milan, Italy). Briefly, the cells were harvested, washed in PBS, and incubated in 100 μL of binding buffer (10 μM Hepes/NaOH pH 7.5, 140 μM NaCl, and 2.5 μM CaCl_2_) containing 1 μL of Annexin-V-FITC (Pharmingen BD) for 15 min in the dark. Then, 100 μL of the same buffer was added to each sample and analyzed by flow cytometry.

### Statistical analysis

When appropriate, T-test and one‐way analysis of variance procedure followed by Dunnett’s multiple comparison test were used to calculate statistical significance. Differences were considered significant when p  ≤  0.05 (*) or highly significant when p ≤  0.0001 (**).

## Results

### Selection of the target CD5 mRNA sequence and synthesis of DNA and PNAs

The CD5 mRNA sequence to be used as the target of our antisense approach was selected as described in the Experimental Section. Since DNA is more nuclease-resistant than RNA [32], to ease the preliminary in vitro hybridization studies, we used the 12-mer DNA sequence having the same sequence of the 12-mer mRNA tract as a model to evaluate the ability of PNA to recognize its target specifically. Next, we synthesized the corresponding 12-mer DNA sequence (**DNA** in Table 1) used for the in vitro hybridization studies. The interaction between the target DNA model and its complementary PNA (**PNA**) or not complementary PNA (**scrambled PNA**) (Table 1) was assessed using non-denaturing polyacrylamide gel electrophoresis (PAGE), thermal difference spectra (TDS), circular dichroism (CD), and CD melting, as reported below.

### Non-denaturing Polyacrylamide Gel Electrophoresis (PAGE)

**Fig 1:**
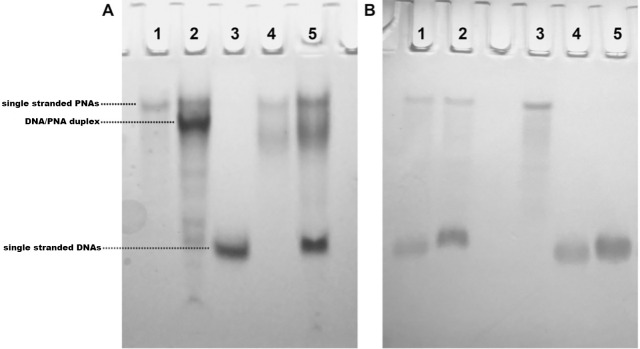
PAGE in 100 mM PBS of: A) **PNA** (lane 1), **DNA** mixed with **PNA** (lane 2), **DNA** (lane 3), **scrambled PNA** (lane 4), and **DNA** mixed with **scrambled PNA** (lane 5); B) **pyrimidine-rich control DNA** mixed with **PNA** (lane 1), **purine-rich control DNA** mixed with **PNA** (lane 2), **PNA** (lane 3), **pyrimidine-rich control DNA** (lane 4), and **purine-rich control DNA** (lane 5). All mixtures were prepared at a 1:3 DNA/PNA ratio.

The ability of the synthesized **PNA** to selectively bind the complementary **DNA** model sequence was first investigated by PAGE analysis (Fig 1). We used the **pyrimidine-rich control DNA**, **purine-rich control DNA**, and **scrambled PNA** sequences as negative controls (Table 1). The PAGE mobility of **DNA/PNA** and **DNA/scrambled PNA** mixtures at the 1:3 molar ratio (Fig 1A, lanes 2 and 5, respectively) was compared to that of **DNA**, **PNA**, and **scrambled PNA** alone (Fig 1A, lanes 3, 1, and 4, respectively). **DNA** and **PNA** migrated as a single band, with the first having a faster migration rate because of the higher negative charge of the sugar-phosphate backbone. The addition of 3 equiv. of **PNA** to **DNA** resulted in the disappearance of the band of free **DNA** and the appearance of a new intense band corresponding to the **DNA/PNA** heteroduplex (Fig 1A, lane 2). Conversely, the addition of 3 equiv. of **scrambled PNA** to **DNA** did not cause the disappearance of the free **DNA**’s band nor the appearance of a new band attributable to the **DNA/scrambled PNA** heteroduplex (Fig 1A, lane 5). Indeed, differently from **PNA**, **scrambled PNA** did not migrate as a single band, but as a couple of bands, the first of which has the same mobility as **PNA**. Though the ESI-MS and HPLC data confirmed the purity and chemical nature of **scrambled PNA**, it is possible that the presence of minor amounts of incomplete scramble PNA could be responsible for the faster band (Fig 1A, lane 4). The binding selectivity of **PNA** for the fully complementary **DNA** sequence was established by the absence of any interaction with the **purine-rich control DNA** (having the complementary purine to pyrimidine ratio than **PNA**) and **pyrimidine-rich control DNA** (having the same pyrimidine to purine ratio than **PNA**), which were chosen as the negative DNA control sequences, as disclosed by the analysis of the PAGE run reported in Fig 1B.

### Thermal Difference Spectra (TDS)

Thermal difference spectroscopy (TDS) provides a simple and reliable technique for identifying nucleic acid secondary structures (e.g., duplex, triplex, quadruplex). Mergny et al. reported the TDS profiles of three duplexes having 100%, 50%, and 0% GC base composition [33]. In particular, the 50% GC-duplex was characterized by a positive diagnostic peak at 267 nm. Considering that our **DNA**/**PNA** complex (42% GC composition) showed the major positive peak at 267 nm, we attributed its TDS profile to that of a **DNA**/**PNA** heteroduplex. On the contrary, no significant peak was found at 267 nm for the **DNA**/**scrambled PNA** mixture (S5 Fig).

### Circular Dichroism (CD) and CD melting

**Fig 2:**
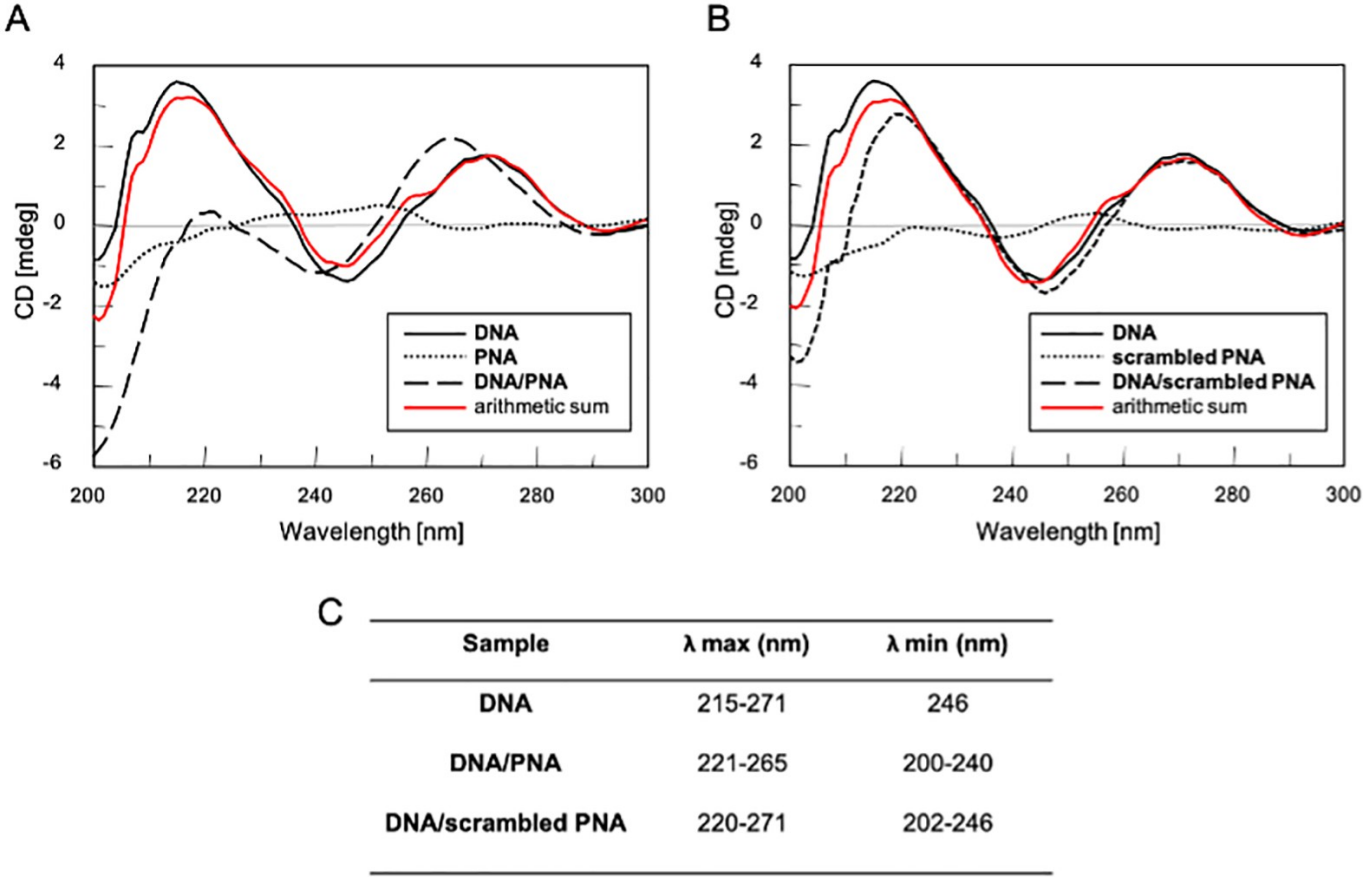
CD profile of the single-strand **DNA** alone (solid black line, panel A and B) and after annealing with **PNA** or **scrambled PNA** (dashed line, A and B respectively); samples were dissolved in 100 mM PBS at pH 6.8. The arithmetic sum of **DNA** and **PNA** or **DNA** and **scrambled PNA** is reported as the red line (panel A and B, respectively). The CD profile of PNA or scrambled PNA alone is reported as the dotted line (panel A and B, respectively). All spectra were acquired at 5°C in the range 200–300 nm and normalized at 300 nm; Table (C) λ values of CD minima and maxima of each sample.

To confirm the formation of the **DNA/PNA** heteroduplex, we recorded the CD profiles of the **DNA/PNA** and **DNA**/**scrambled PNA** mixtures (1:3 molar ratio) (dashed lines in Fig 2, panel A and B, respectively) in comparison with the CD spectra of **DNA** (solid black lines), **PNA** (dotted line, panel A) and **scrambled PNA** (dotted line, panel B) alone. The **DNA/PNA** spectrum showed the characteristic CD profile of an antiparallel DNA/PNA heteroduplex, characterized by two positive Cotton’s effects around 220 nm and 260 nm and two negative Cotton’s effects around 200 and 240 nm [14]. Conversely, the absence of the typical CD heteroduplex profile and the complete overlapping of the experimental CD spectrum of **DNA/scrambled PNA** with the arithmetic sum of the individual spectral components (red line) confirmed that the **DNA** probe does not bind the not-complementary **scrambled PNA**. The analysis of the CD profile of the **DNA/PNA** complex showed a higher value of CD absorbance than the arithmetic sum of two components (red line, Fig 2 panel A). We attributed this finding to the formation of stacking interactions between different planes of the **DNA/PNA** heteroduplex. The binding interaction was further confirmed by the 6 nm blue shift observed for the longer wavelength positive CD maximum, centered at 265 nm rather than at 271 nm (Fig 2, panel C). To investigate the thermal stability of the **DNA/PNA** heteroduplex, we performed a CD melting study (S6 Fig). The resulting melting temperature (Tm) was found to be 37°C. This data supported the suitability of the herein proposed PNA-based antisense approach at the physiological body temperature, also considering that the Tm of PNA/RNA heteroduplexes is generally higher than that of the corresponding **PNA/DNA** heteroduplexes [15].

### Intracellular delivery of CD5 PNA

**Fig 3:**
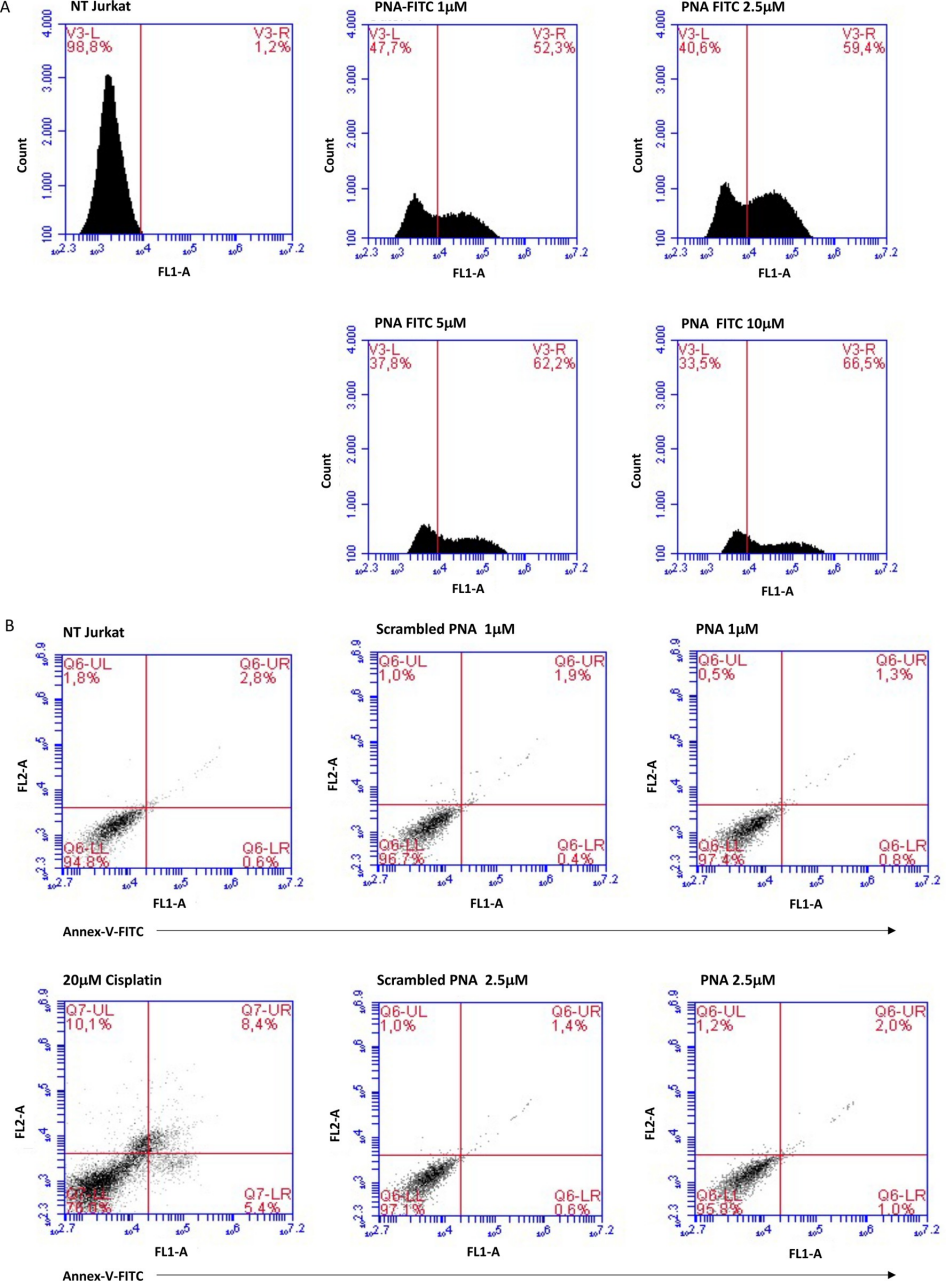
Evaluation of PNA transfection efficiency and cell death analysis in Jurkat cells. (A) Flow cytometric histograms of Jurkat cells transfected with **PNA-FITC** to measure PNA delivery efficiency into the cells. Different concentrations of **PNA-FITC** were used (1, 2.5, 5, and 10 μM), and 48 h after transfection the cells were harvested and analyzed by flow cytometry. A marker was placed on non-transfected cells (NT) and FL1 fluorescence measured as percentage of cells uptaking the PNA. The figure shows two peaks on transfected cells that can be interpreted as the positive (V3-R) and negative (V3-L) datasets. (B) Flow cytometric evaluation of cell death. Two different concentrations (1.0 and 2.5 μM) of **PNA** or **scrambled PNA** were used for transfection. Necrotic and apoptotic cells were detected by annexin V and PI staining followed by flow cytometry analysis 48 h after transfection. The LR, UR, and UL quadrants measure the cells Annexin V^+^/PI^−^(early apoptosis), Annexin V^+^/PI^+^ (late apoptosis), and Annexin V^–^/PI^+^ (necrosis), respectively. The LL quadrants represent the percentage of double-negative cells. No variation in Annexin V and PI percentage was observed in **PNA**-treated cells compared to NT, or cells treated with the **scrambled PNA**. In contrast, the treatment with 20 μM cisplatin for 24 h used as cell death positive control, induced Jurkat cell death as expected.

To examine the cellular delivery of the PNA directed against the CD5 mRNA, we transfected Jurkat cells with different concentrations of the **PNA-FITC** and **scrambled PNA-FITC.** The PNA delivery was analyzed by flow cytometry 48 h after transfection. The optimal conditions of PNAs transfection were evaluated by quantifying the background-fluorescence in non-transfected cells used as negative control (NT Jurkat in Fig 3A). As shown in Fig 3A, the PNA transfection efficiency was measured by evaluating the FL1-A fluorescence corresponding to the uptake of **PNA-FITC**. The percentage of PNA intracellular delivery was 52.3%, 59.4%, 62.2% and 66.5% at the concentration of 1 *μ*M, 2.5 μM, 5 μM and 10 μM PNA, respectively. No variations in delivery efficiency were observed with scrambled PNA (data not shown). Since a reduction of the total cell number without a notable increase in transfection efficiency was observed at higher PNA concentrations (5 μM and 10 μM), we chose to use PNA concentrations of 1 μM and 2.5 μM for the subsequent experiments. To assess whether PNA transfection can cause cellular toxicity at the selected doses, we evaluated the cell death of Jurkat cells transfected with 1 μM and 2.5 μM **PNA** or **scrambled PNA** 48 h after transfection (Fig 3B). Cells were stained with annexin V and PI and subsequently analyzed using flow-cytometry to differentiate between necrosis and early and late apoptosis. The dual negative staining of transfected cells demonstrated that PNA transfection does not affect cell viability. Therefore, no significant side effects were observed in these cells.

### CD5 expression is down-modulated by PNA treatment

**Fig 4:**
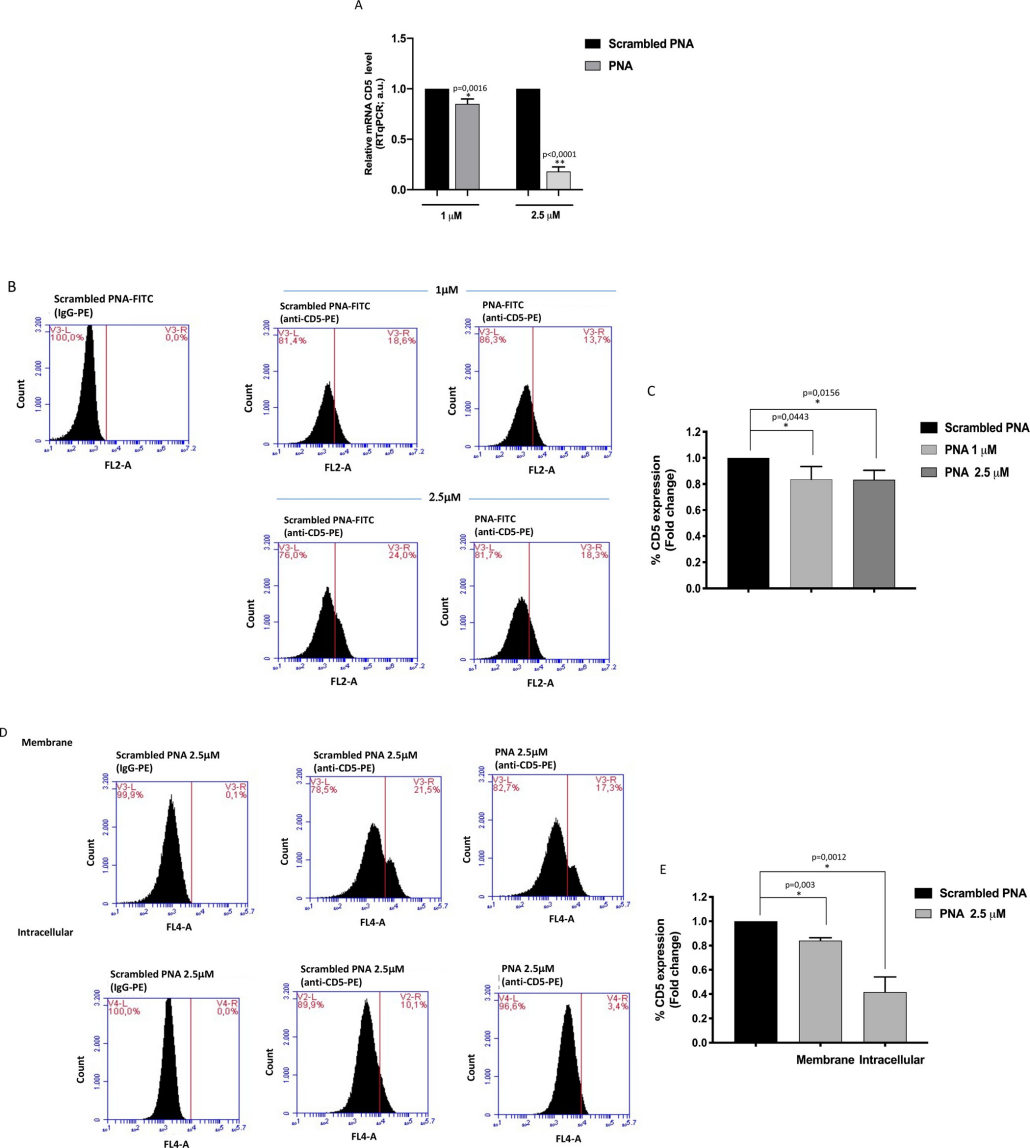
Evaluation of PNA effect on CD5 expression. Jurkat cells were transfected with two different concentrations of **scrambled PNA** or **PNA** (1.0 and 2.5 μM). (A) Evaluation of CD5 mRNA expression levels was performed by RT-qPCR 48 h after transfection by comparing **PNA** transfected cells to cells transfected with **scrambled PNA** at both concentrations. Cells transfected with **PNA** showed a decreased CD5 mRNA expression, differently from cells transfected with **scrambled PNA** used as negative control. HPRT was used as a reference gene for the relative normalization of gene expression analysis. The data shown are the mean ± SD of three independent experiments. Differences were considered significant when p ≤ 0.05 (*) and p ≤ 0.0001 (**) versus **scrambled PNA**. (B) Flow cytometric histograms of membrane CD5 expression in Jurkat cells transfected for 48 h with **PNA-FITC** or the **scrambled PNA-FITC**. A marker was placed on the control isotype IgG stained cells and FL2 fluorescence measured as percentage of CD5 positive cells. (C) Data are shown as fold change of CD5 expression of Jurkat cells transfected with **PNA-FITC** at 1.0 and 2.5 μM, each of them compared to the corresponding **scrambled PNA-FITC**, used as control (arbitrary value = 1). Values are the mean ± SD of three independent experiments. (D) Flow cytometric histograms of membrane and intracellular CD5 expression in Jurkat cells transfected for 48 h with 2.5 μM **PNA-FITC** or the control **scrambled PNA-FITC**. A marker was placed on the respective control isotype IgG stained cells and FL2 fluorescence measured as percentage of CD5 positive cells. (E) Data are shown as fold change of membrane and intracellular CD5 expression in Jurkat cells transfected with **PNA-FITC**, each of them compared to the corresponding **scrambled PNA-FITC**, used as control (arbitrary value = 1). Values are the mean ± SD of three independent experiments. Differences were considered significant when p ≤ 0.05 (*) versus **scrambled PNA**.

We next examined the ability of **PNA** to target CD5 mRNA specifically. To this aim, Jurkat cells were transfected with two different concentrations of **PNA** or **scrambled PNA** as negative control and then analyzed for CD5 mRNA expression levels by quantitative reverse transcription PCR (RT-qPCR). As shown in Fig 4A, **PNA** treatment significantly down-regulated CD5 mRNA levels at both concentrations, even though more efficiently at 2.5 μM. Next, we confirmed the reduction of CD5 expression at the protein level by flow cytometry. We initially measured the membrane-bound CD5 levels in Jurkat cells transfected with **PNA-FITC** and **scrambled PNA-FITC**. FITC positive cells were gated to assess CD5 expression only in the transfected cells. The percentage of CD5+ cells was found reduced of 16% in cells transfected with **PNA-FITC** at both PNA concentrations tested, although with no consistent dose-dependent effect (Fig 4, panels B, C). To better elucidate the effect of PNA treatment on CD5 protein levels in Jurkat cells, we then separately evaluated membrane-bound and intracellular CD5 expression levels. In this case, cells were transfected with the higher PNA dose (2.5 μM) according to the more dramatic reduction in mRNA levels detected at this concentration (Fig 4A). Positive cell percentages for membrane or intracellular CD5 proteins are shown in Fig 4D and 4E. Results indicate a more significant reduction in intracellular CD5 expression (59%) with respect to the membrane fraction (16%) following **PNA** transfection. We speculate that the stronger reduction of intracellular CD5 in comparison to membrane-bound CD5 can be related to the slow turnover rate of surface CD5 protein [34]. Nevertheless, a reduction of about 75% in the total CD5 protein expression (membrane plus intracellular fractions) was found following PNA treatment (see Fig 4E). Our results prove that the negative regulatory effects of the PNA treatment are directly related to impaired CD5 mRNA function and, consequently, to reduced protein levels.

### Evaluation of CD5 PNA treatment on PBMCs from B-CLL patients

**Fig 5:**
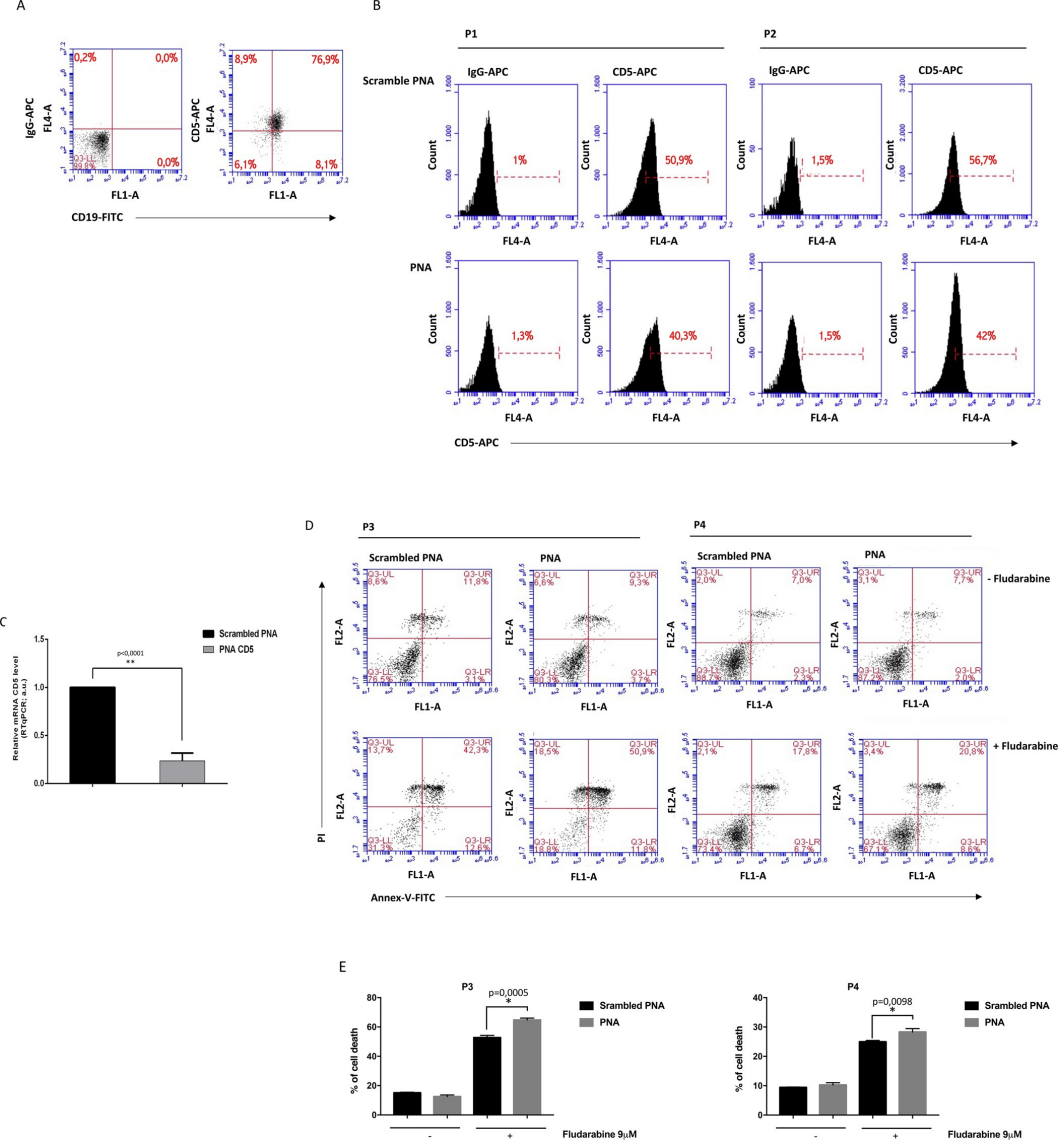
PNA impairs CD5 expression in B-CLL cells and sensitizes to fludarabine-induced cell death. (A) Representative dot plots of PBMCs characterization for the expression of CD19 and CD5. (B) Flow cytometric analysis of CD5 expression in PBMCs from two B-CLL patients (P1 and P2) transfected with 1 μM **scrambled PNA** or **PNA**. A marker was placed on the relative control isotype IgG stained cells and FL4 fluorescence measured as percentage of CD5 positive cells. To evaluate CD5 reduction, for each patient the ratio between the percentage values of PNA and scrambled PNA subtracted of their corresponding IgG background (values shown in each panel) was calculated. The obtained scrambled PNA percentage was set as 100. The ratio values for P1 and P2 were 78% and 73%, respectively, corresponding to a reduction of 22% and 27% (average reduction of about 25%). (C) **PNA** transfection significantly decreased CD5 transcript levels compared to **scrambled PNA** in PBMCs from three CLL patients (P1, P3, and P5). p ≤ 0.0001 (**). (D) Representative flow cytometric histograms of Annexin V/PI staining. PBMCs from two B-CLL patients (P3 and P4) transfected with 1 μM **scrambled PNA** or **PNA** were incubated in the absence (-fludarabine, top panels) or presence (+ fludarabine, bottom panels) of 9 μM fludarabine for 72 h. (E) Histograms showing cell death values (Annexin V^+^/PI^−^cells and Annexin V^+^/PI^+^ cells) obtained from PBMCs of patients P3 and P4. Data shown represent the mean ± SD from technical triplicates.

PBMCs purified from peripheral blood of B-CLL patients were characterized for the presence of CD5+CD19+ B-cells, typically enriched in B-CLL patients (Fig 5, panel A). Given the limited amount of PBMC recovery from each patient, each cell sample was sufficient for only one or at most two different experiments. Since the 1 μM PNA concentration had resulted effective in substantially reducing CD5 mRNA levels (Fig 5, panel C), we used this lower effective dose in primary PBMCs.

Firstly, we evaluated membrane-bound CD5 protein levels in PBMC cells transfected with **PNA** or **scrambled PNA** by flow cytometry. Results showed that **PNA** specifically down-modulates cell membrane-CD5 levels in B-CLL patients (Fig 5, panel B). In detail, in PBMC from two B-CLL patients (P1 and P2) we observed an average reduction of about 25% of membrane-bound CD5 protein levels following PNA treatment with respect to cells treated with scrambled PNA. Furthermore, we observed that CD5 mRNA levels were decreased of 77% in B-CLL PBMCs compared to the scrambled control (Fig 5, panel C). To examine if CD5 downmodulation can enhance sensitivity to chemotherapy, CLL PBMCs from patients P3 and P4 transfected with **PNA** or **scrambled PNA** were treated with the chemotherapic drug fludarabine. Then apoptosis was evaluated by annexin V/PI assay (Fig 5, panel D). Consistent with results in Jurkat cells (Fig 3B), **PNA** alone does not induce cell death whereas, interestingly, in combination with fludarabine, **PNA** treatment significantly increased the percentage of apoptotic cells (22.5% in P3 and 13% in P4), also supporting the physiological significance of PNA-dependent CD5 down-modulation in CLL. Collectively, these results show that **PNA** treatment reduces CD5 mRNA and protein levels in PBMCs from B-CLL patients and sensitizes B-CLL cells to chemotherapy-induced cell death.

## Discussion

Recently, CD5 has been proposed as a promising therapeutic target in CLL. Given the severity of the disease and the importance of the newly emerged roles of CD5, great efforts are being made to develop novel therapeutic approaches to inhibit the oncogenic potential of the aberrant CD5 signaling. PNAs are oligonucleotides analogs characterized by high resistance to nucleases and proteases, excellent chemical and biological stability, and high hybridization affinity and sequence specificity toward DNA and RNA. Many studies have recently addressed the therapeutic potential of PNAs in gene modulation in several disease models. In this paper, we synthesized a suitable PNA molecule (**PNA**) to investigate its ability to bind the target sequence on the CD5 mRNA and effectively downmodulate CD5 expression in CLL. Initially, a high **DNA/PNA** complex’s stability was measured, allowing to expect that this PNA molecule could hybridize with higher affinity to its mRNA target. To explore the effectiveness of PNA treatment, we chose to use the human T-leukemia Jurkat cell line due to its high and more stable expression of CD5 compared to the few commercially available B-CLL cell lines [35, 36]. We obtained an appreciable intracellular delivery efficiency with cationic liposomes by introducing two negative charges on FITC-labelled PNA oligomers without significantly affecting cell viability. Following **PNA** treatment, Jurkat cells showed reduced CD5 levels both at RNA and protein levels. It is to be noted that, although more evident at the mRNA level, the total CD5 protein levels (membrane-bound plus intracellular fractions), were also consistently down-modulated by PNA treatment. These results suggest that PNA-mediated mechanisms may involve inhibition of translation and possibly, as a consequence of translation inhibition, mRNA degradation [37]. Accordingly, we observed a reduction of both membrane-bound and intracellular CD5 fractions.

Interestingly, similar results on CD5 down-modulation triggered by **PNA** treatment were obtained in peripheral blood mononuclear cells from B-CLL patients, representing a more physiological setting than immortalized cell lines. Based on the evidence that CD5 acts as a prosurvival factor in B-CLL cells [38, 39], we hypothesized that CD5 down-regulation could represent a mechanism to increase cell sensitivity to pro-apoptotic stimuli. To verify this hypothesis, cells were co-treated with PNA and fludarabine, a drug commonly used in CLL causing inhibition of DNA synthesis and, consequently, induction of apoptosis [40]. Our results showed that PNA treatment enhanced the fludarabine-induced apoptosis.

In this context, the unique physiochemical properties of PNAs, including the prolonged half-life due to resistance to nuclease and protease-mediated degradation, their strong binding efficiency and low toxicity, make them attractive tool for the development of antisense and antigene strategies. On the other hand, their limited uptake in living cells has hampered the progress of their use for regulating gene expression and prompted the development of modified PNA structures and delivery strategies [41, 42]. In our hands, the presence of two negative charges on the PNA oligomers and the use of a cationic transfectant reagent (Lipofectamine) resulted in appreciable PNA uptake. Finally, it is also to be noted that although also expressed in normal T-cells, in the context of B-lymphocytes, aberrant expression of CD5 is restricted to malignant B cells. This unique feature provides a strong incentive to develop novel drug delivery nanoparticles bearing PNAs as the cargo and decorated with molecular moieties targeting specific surface B-CLL markers. This would maximize the amount of PNAs reaching the target tumor cells, with minimum drug leakage into normal cells.

## Conclusion

Our results pave the way to further studies to evaluate CD5 PNA treatment’s feasibility in combination with chemotherapy as an appealing approach for more effective therapeutic strategies in B-CLL.

## Supporting information

S1 Fig Expansion of the ESI-MS spectrum of DNA (5’-TTGGGAGAGAAA-3’) recorded in the negative ion mode. Calcd. for [M– 4H]^–4^ 938.2, found 938.2.
(PDF)

S2 Fig Expansions of the ESI-MS spectra of **PNA** (A) and **scrambled PNA** (B) recorded in the positive ion mode. Calcd. for [M + 3H]^3+^ 1201.4, [M + 4H]^4+^ 901.3, [M + 5H]^5+^ 721.3.
(PDF)

S3 Fig Not processed image of PAGE reported in Fig 1A. (PDF)

S4 Fig Not processed image of PAGE reported in Fig 1B. (PDF)

S5 Fig Overlapped TDS spectra of DNA annealed with PNA (blue line) or scrambled PNA (green line). (PDF)

S6 Fig CD melting curve of DNA/PNA mixture at 1:3 ratio obtained by monitoring the absorbance at 265 nm. Heating rate: 1°C/min.
(PDF)

S1 Raw images (PDF)
