# Supplementary material for: Exploring a peptide nucleic acid-based antisense approach for CD5 targeting in chronic lymphocytic leukemia
Source: PLoS One. 2022 Mar 31;17(3):e0266090. doi: 10.1371/journal.pone.0266090 (PMC8970396; doi:10.1371/journal.pone.0266090)
Supplement: S1 Fig — Calcd. for [M– 4H]–4 938.2, found 938.2. (PDF) [file pone.0266090.s001.pdf]

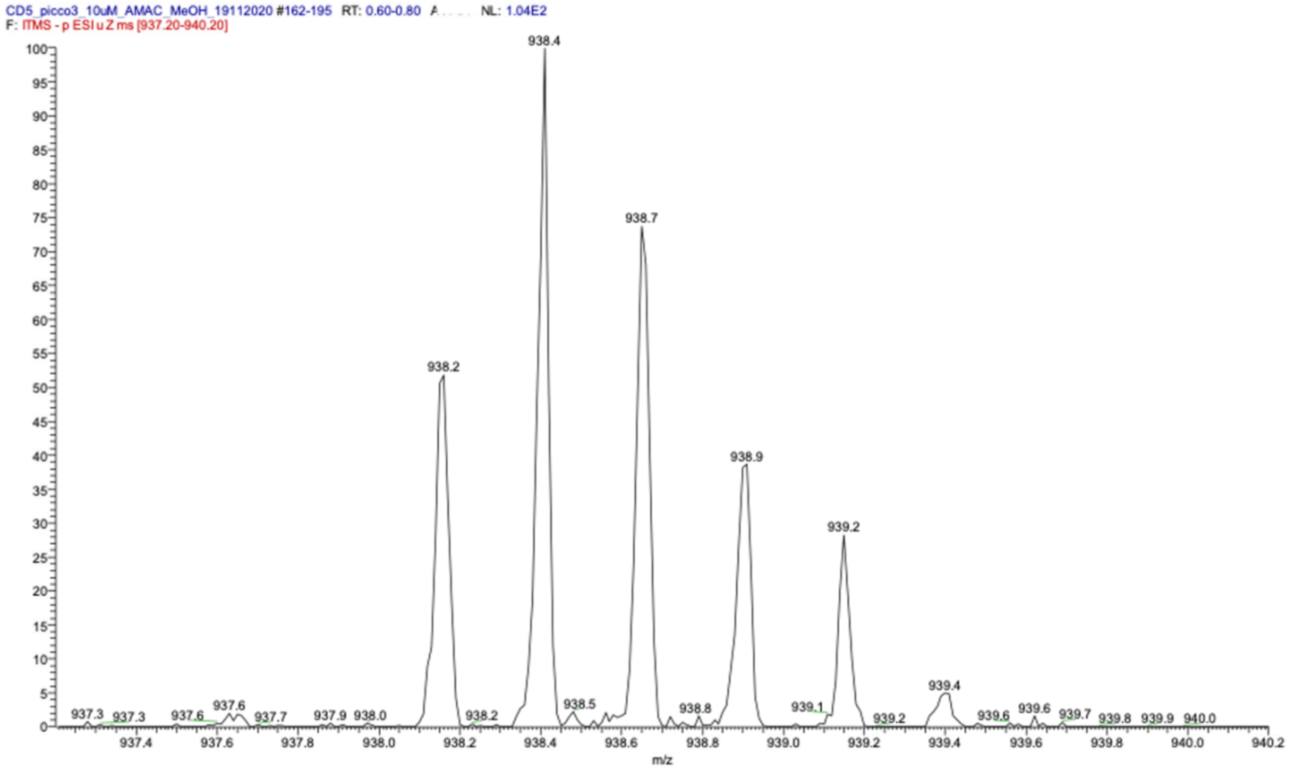

S1 Fig. Expansion of the ESI-MS spectrum of **DNA** (5'-TTGGGAGAGAAA-3') recorded in the negative ion mode. Calcd. for $[M – 4H]^{–4}$ 938.2, found 938.2.
