# Supplementary material for: Exploring a peptide nucleic acid-based antisense approach for CD5 targeting in chronic lymphocytic leukemia
Source: PLoS One. 2022 Mar 31;17(3):e0266090. doi: 10.1371/journal.pone.0266090 (PMC8970396; doi:10.1371/journal.pone.0266090)
Supplement: S2 Fig — Expansions of the ESI-MS spectra of PNA (A) and scrambled PNA (B) recorded in the positive ion mode. Calcd. for [M + 3H]3+ 1201.4, [M + 4H]4+ 901.3, [M + 5H]5+ 721.3. (PDF) [file pone.0266090.s002.pdf]

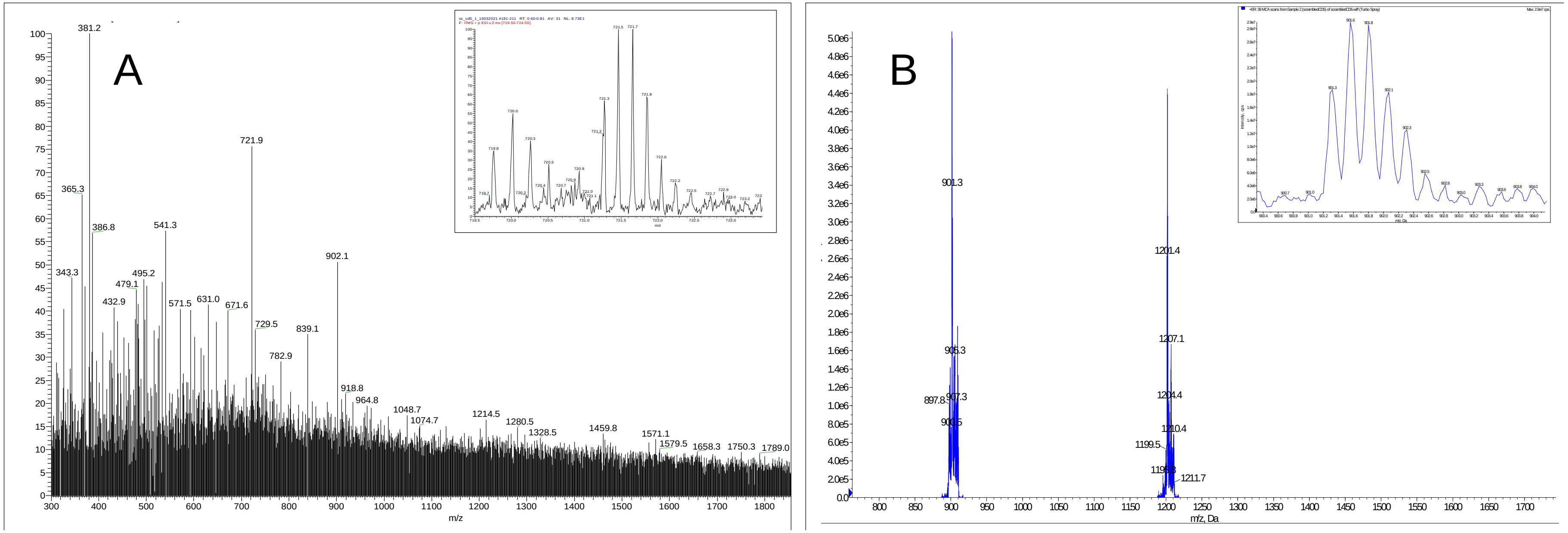

S2 Fig. Expansions of the ESI-MS spectra of **PNA** (A) and **scrambled PNA** (B) recorded in the positive ion mode. Calcd. for $[M + 3H]^{3+}$ 1201.4, $[M + 4H]^{4+}$ 901.3, $[M + 5H]^{5+}$ 721.3.
