# Supplementary figures and images for: Exploring a peptide nucleic acid-based antisense approach for CD5 targeting in chronic lymphocytic leukemia
Source: PLoS One. 2022 Mar 31;17(3):e0266090. doi: 10.1371/journal.pone.0266090 (PMC8970396; doi:10.1371/journal.pone.0266090)

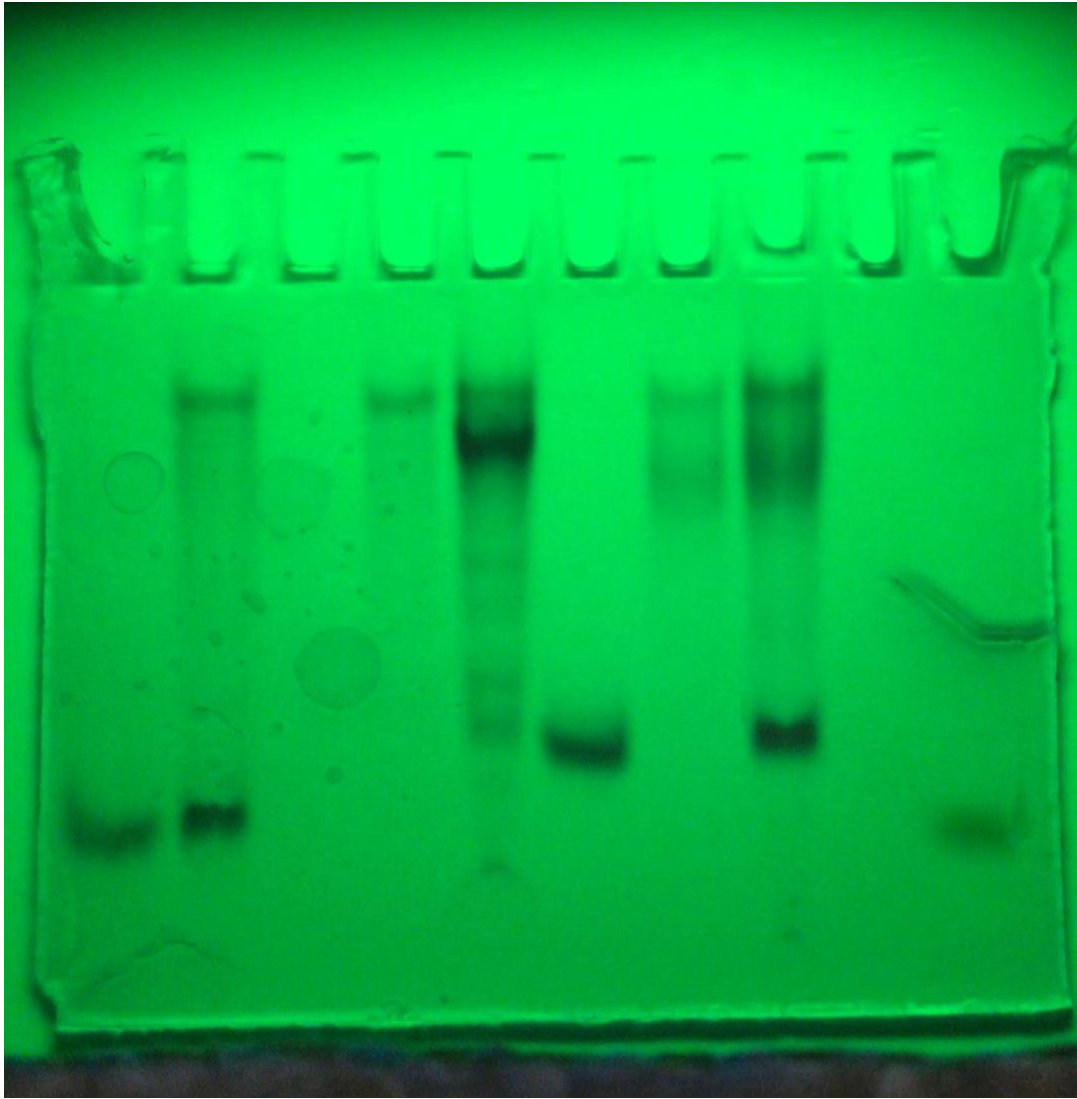

**S3 Fig.** Not processed image of PAGE reported in Fig 1A.

Supplement: S3 Fig — (PDF) [file pone.0266090.s003.pdf]

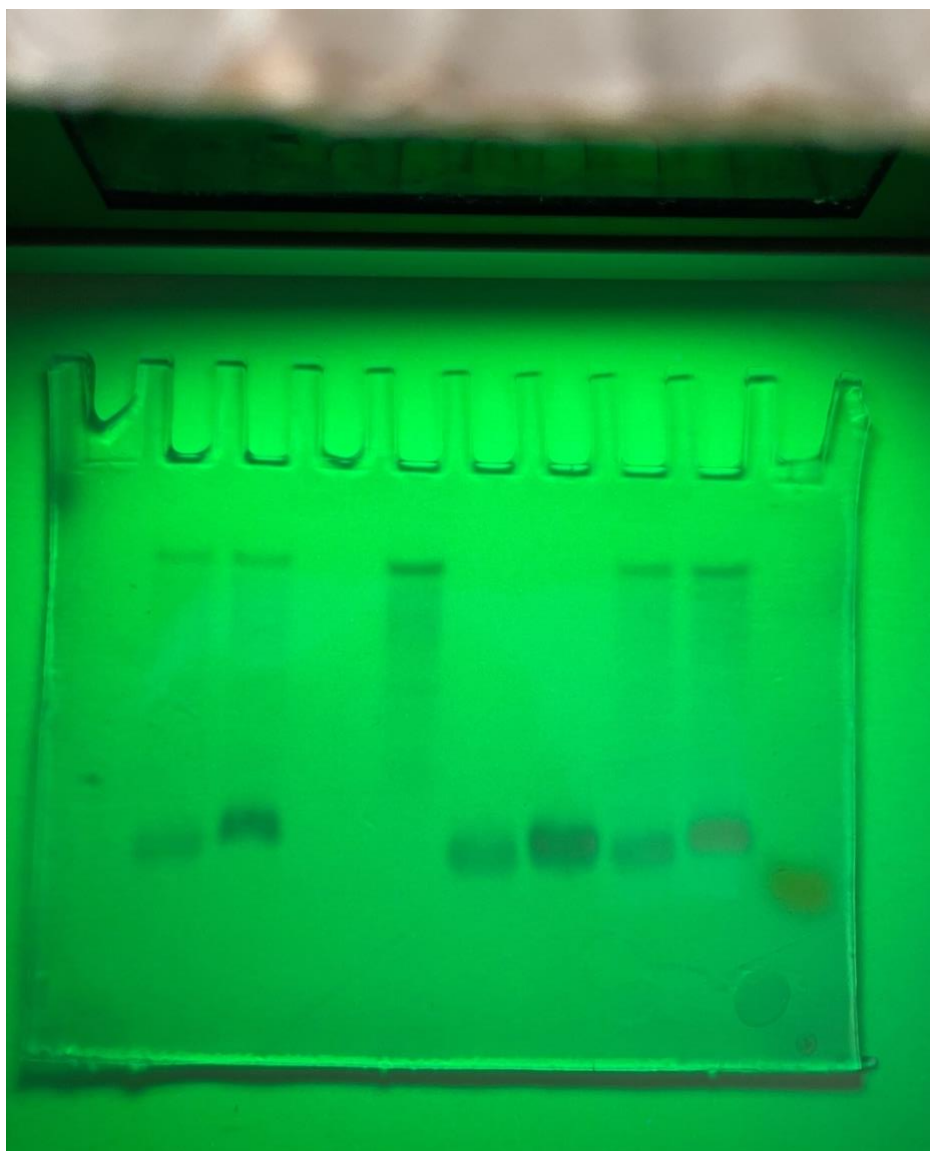

**S4 Fig.** Not processed image of PAGE reported in Fig 1B.

Supplement: S4 Fig — (PDF) [file pone.0266090.s004.pdf]

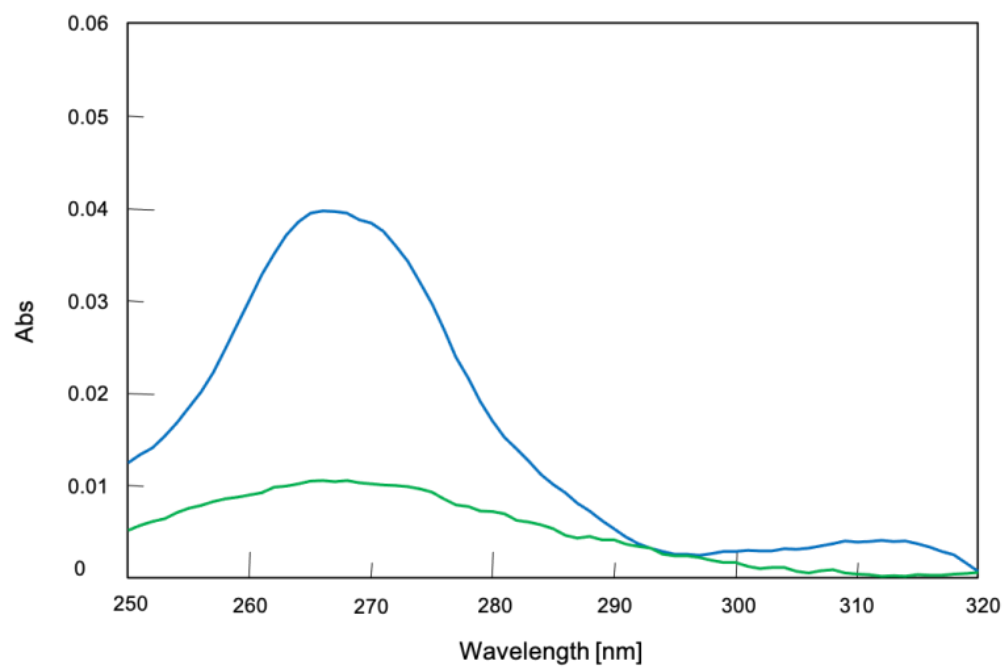

**S5 Fig.** Overlapped TDS spectra of DNA annealed with PNA (blue line) or scrambled PNA (green line).

Supplement: S5 Fig — (PDF) [file pone.0266090.s005.pdf]

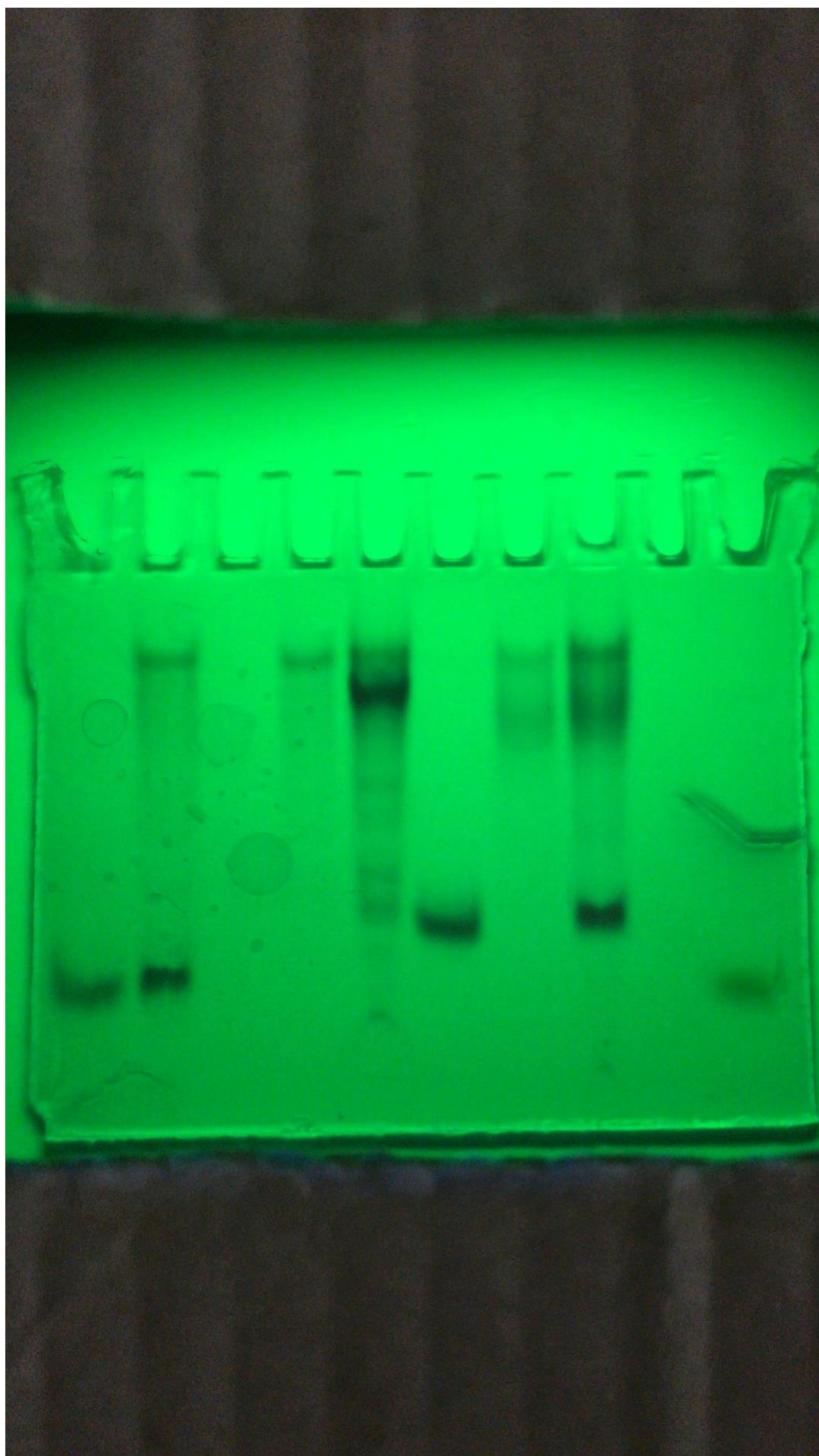

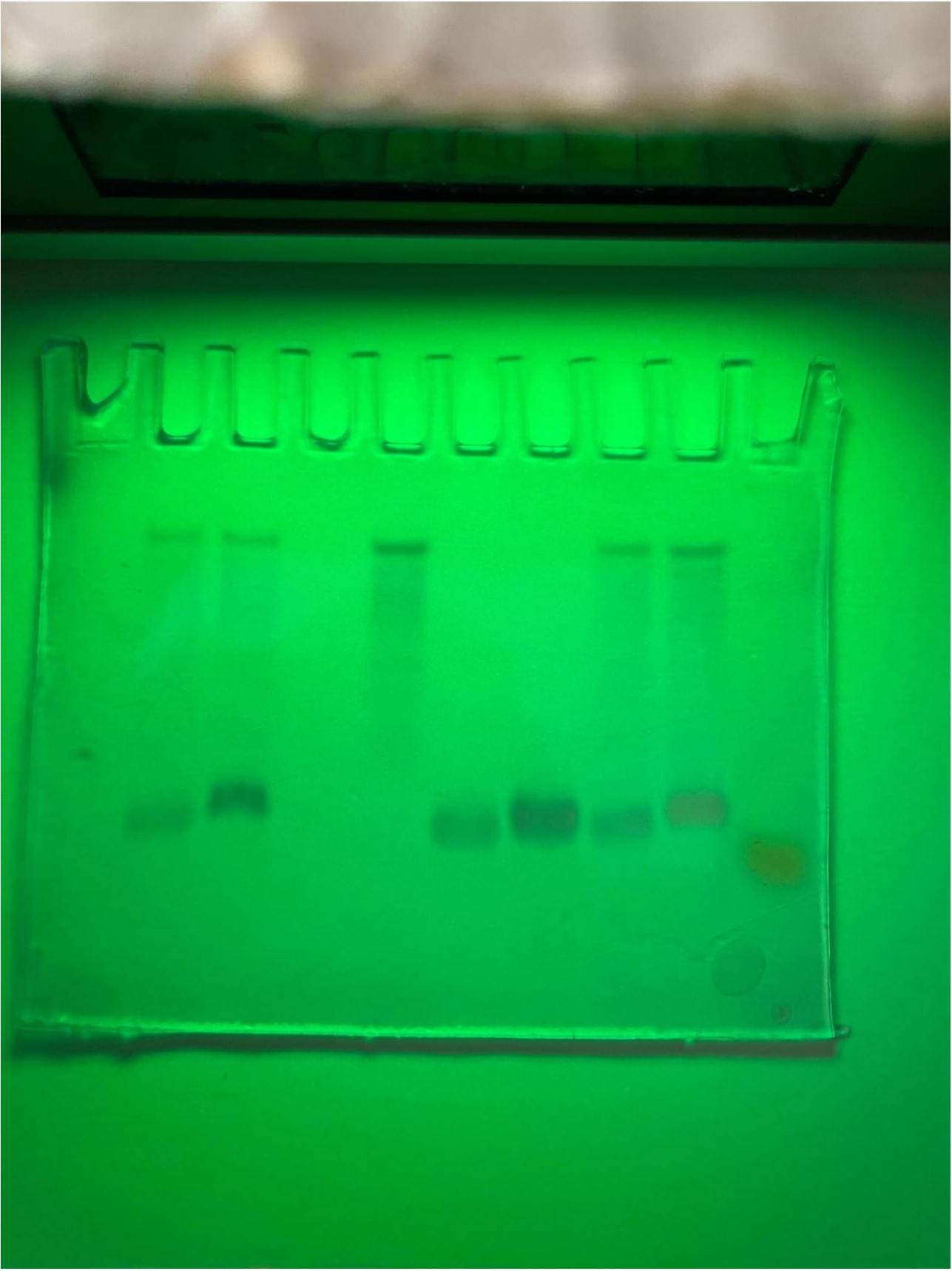

Supplement: S1 Raw images — (PDF) [file pone.0266090.s007.pdf]
