# Supplementary material for: Exploring a peptide nucleic acid-based antisense approach for CD5 targeting in chronic lymphocytic leukemia
Source: PLoS One. 2022 Mar 31;17(3):e0266090. doi: 10.1371/journal.pone.0266090 (PMC8970396; doi:10.1371/journal.pone.0266090)
Supplement: S6 Fig — Heating rate: 1°C/min. (PDF) [file pone.0266090.s006.pdf]

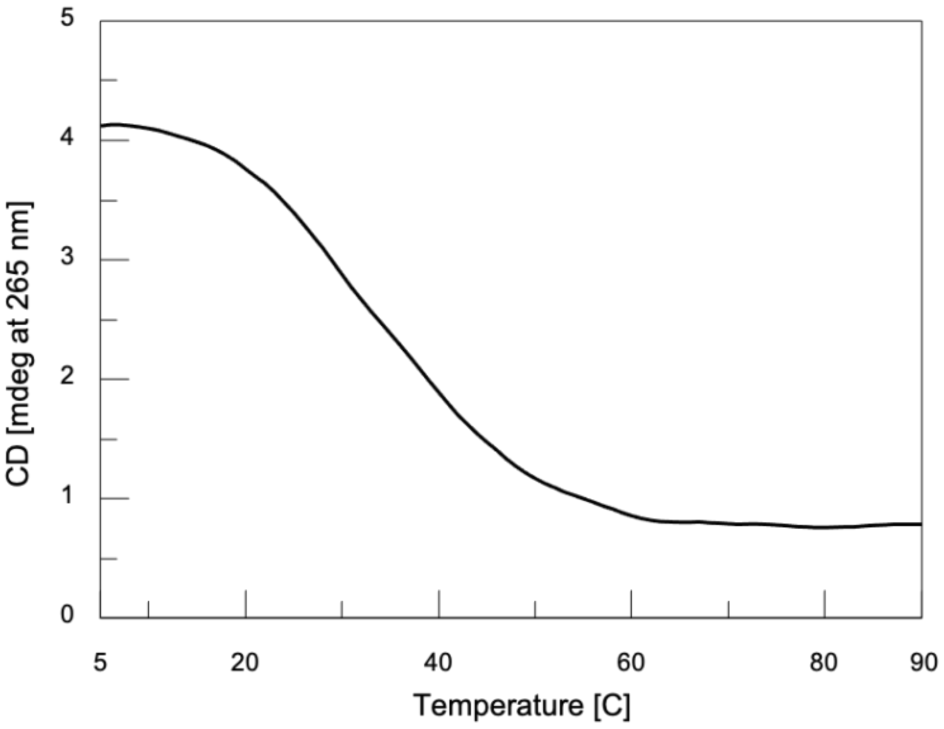

Sample	Tm
**DNA/PNA**	37 °C

S6 Fig. CD melting curve of DNA/PNA mixture at 1:3 ratio obtained by monitoring the absorbance at 265 nm. Heating rate: 1 °C/min.
